# Altruism and the evolution of resource generalism and specialism

**DOI:** 10.1002/ece3.206

**Published:** 2012-03

**Authors:** Michael B Bonsall, Alison E Wright

**Affiliations:** Department of Zoology, University of Oxford,South Parks Road, Oxford OX1 3PS, United Kingdom

## Abstract

The evolution of resource specialism and generalism has attracted widespread interest. Evolutionary drivers affecting niche differentiation and resource specialization have focused on the role of trade-offs. Here, however, we explore how the role of cooperation, mediated through altruistic behaviors, and classic resource–consumer dynamics can influence the evolution of resource utilization. Using an evolutionary invasion approach, we investigate how critical thresholds in levels of altruism are needed for resource specialization to arise and be maintained. Differences between complementary (essential) and substitutable resources affect the evolution of resource generalists. The strength of resource preferences coupled with the levels of altruism are predicted to influence the evolution of generalism. Coupling appropriate evolutionary game and ecological dynamics lead to novel expectations in the feedbacks between social behaviors and population dynamics for understanding classic ecological problems.

## Introduction

Defining the fundamental, and more importantly, the realized niche of species is an ecological problem that continues to be the focus of considerable effort. As is widely appreciated, Hutchinson (1957) defined an ecological niche as a *n*-dimensional hypervolume that delimits patterns of resource utilization and exploitation along a set of ecological axes. This theory of niches has generally been developed from the basis that species interact and compete for shared essential resources. The outcome of this interspecific competition is well known: species coexistence is not expected unless the interaction amongst conspecifics outweighs the interaction amongst heterospecifics and the number of species that co-occur cannot exceed the number of resources. While arguably tautological (May 1973), Levin (1970) and others have extended this idea to consider broader limiting factors (rather than simply focusing on resources) and reached a more generic prediction: coexistence is not possible if the number of species are limited by less than the equivalent number of limiting factors. In spite of caveats on the extent of resource use by consumers, limiting factors act to define niche axes and the niche width defines the degree of variability in resource utilization by a species: narrow niche widths and hence small variation in resources used is expected to correlate with resource specialism while species with broad niche widths (large variation) are resource generalists (Roughgarden 1972).

Determining the evolution of specialists or generalists has focused primarily on the assessment of fitness on multiple resources. Levins (1962, 1968) argued that while fitness on multiple resources is generally negatively correlated—performance on one resource occurs at a cost of performance on a second resource—the shape of this trade-off is important. The expectation is that consumers are generalists when equally well adapted to multiple resources and the fitness is concave outwards such that small changes in diet have small incremental changes on fitness. In contrast, consumers are expected to be specialists if the fitness function is concave inwards and small changes in resource use have large fitness consequences. As noted, fundamental to resource specialization is this necessity for trade-offs (Futuyma and Moreno 1988). Phenotypic trait trade-offs are, therefore, putatively associated with negative genetic covariance (e.g., Leroi et al. 1994). However, the evolution of specialization might not necessarily require negative genetic correlations (Fry 1996; Whitlock 1996). Specialization can be favored if genotypes that are under positive selection on one resource are under weaker positive selection or are neutral on alternative resources (Fry 1996; Dykhuizen and Dean 2004). Levins’ fitness set approach further assumes that preferences (if not performance) for different resources is equal. This idea of diet breadth has been the focus of considerable work as the driver of generalism and specialism (e.g., MacArthur and Pianka 1966; Schoener 1974; Abrams 2006). Resources have been widely classified into different types: substitutable, essential, complementary, switching, or antagonistic (Tilman 1982) and have important implications for the structure of ecological communities. Explicit consideration of resource types is known to influence competition (Leon and Tumpson 1975); for example, essential resources affect the outcome of interspecific competition by altering the strength of species interactions (Abrams 1987). These diet classifications clearly have the potential to influence the evolution of resource utilization patterns. Furthermore, additional ecological attributes, in particular the role of environmental heterogeneity, are known to affect species coexistence (Armstrong and McGehee 1980) and coupled with diet allow alternative conditions for the maintenance of specialism and generalism to be derived (Wilson and Yoshimura 1994; Abrams 2007; Debarre and Gandon 2010).

Processes that affect the maintenance of polymorphisms will have an influence on the patterns of resource utilization. Haldane (1931, 1932) argues that intense competition among individuals will lead to a variable response to the environment rather than a higher average response and it is well known that this affects the conditions for the maintenance of polymorphisms (tacitly assumed to be generalists). Furthermore, the maintenance of polymorphism is determined by local population structures, heterozygote advantage, and mutation-selection balance (Karlin and Campbell 1981; Maynard–Smith 1998). For instance, instability of niche polymorphism (and hence the evolution of specialists) is expected under population structures associated with hard selection (selection dependent only on gene frequencies) as particular genotypes are favored and fixed in a population. Local population structure and soft selection (where density and frequency-dependent selection for genotypes operates) can act to maintain genetic variation (Wilson and Turelli 1986) and promote generalism.

The ability to access resources requires appropriate morphological and physiological adaptations (Futuyma and Moreno 1988). These properties influence an organism’s ability to select appropriate habitats and/or resources and, consequently affect niche width. The ability to access resources may require additional behavioral adaptations such as cooperative help from conspecifics. Broadly, such cooperative actions are behaviors that confer a benefit to an individual at a cost to another (Trivers 1985). In succinct style, Haldane (1932; pp. 207–210) illustrates how cooperation in groups might spread provided most individuals in a group behave altruistically. Hamilton (1963) extended this argument to show that the behavioral diversity observed among species, as well as morphological adaptations, are both products of evolution, and that this evolution can arise through altruistic behaviors such that a behavior spreads if the fitness gain is at least twice the loss. However, the consequences of this class of behaviors on the evolution of resource specialism are poorly understood even though collective altruistic behaviors are implicated in the successful exploitation of resources (Dugatkin 1997). For example, female bark beetles (*Dendroctonus montanus*) use aggregation pheromones to call in conspecifics to overcome tree defenses (Wyatt 2003). As female density increases, individuals have higher reproductive successes in terms of pupae per attack (until the effects of intraspecifc competition increase in severity) (Raffa and Berryman 1983; Berryman et al. 1985). Cooperative actions have also been implicated in the capture of prey by social spiders (Whitehouse and Lubin 2005). The social spider, *Anelosimus eximius*, builds communal webs and group foraging allows capture of prey of increasing size. This offsets the decline in the number of prey caught per individual as web size and hence colony size increases. Maximum resource intake occurs at intermediate spider densities (Yip et al. 2008). Individual success rates in lion foraging are also correlated with ecological attributes such as prey type and group structure (Scheel and Packer 1991). Groups of lions have higher success foraging than individuals (Dugatkin 1997) but this is open to exploitation by lions that refrain to engage in specific hunting bouts (Scheel and Packer 1991). The exploitation of hosts by opportunistic pathogenic bacteria, such as *Bacillus thuringiensis,* involves sharing the exploits of toxin production from multiple individuals as single individuals are incapable of overcoming host defenses. The exploitation of toxin producers by cheats (nontoxin producing strains) has consequences for pathogen virulence (Raymond et al. 2007, 2009), host-pathogen epidemiology (Bonsall 2010) and the evolution of pathogen strain specificity (Berry et al., unpubl. ms.).

While the evolutionary biology of social interactions is well established (see Bourke 2011 for a recent overview), the broader ecological outcome of altruistic behaviors on the effects of interspecific competition and community organization are unclear. Here, our aim is to redress this and explore the consequence of altruism on the evolution of resource specialization and generalism. The approach we develop combines an evolutionary game with ecological dynamics: by coupling replicator dynamics (describing the proportion of altruists in a consumer population) with ecological resource–consumer dynamics, we aim to explore how greater access to resources manifest through altruistic acts can influence the evolution of resource specialization and generalism. Importantly, an evolutionary invasion approach is used to determine conditions that favor the origin over the maintenance of specialists and generalists by exploring the expected outcome of different resource utilization patterns when altruism operates to increase resource acquisition rate. In the next section, we begin by introducing the model framework, laying out the evolutionary dynamics of altruism, and the ecological dynamics of the resource–consumer interaction. We show how different levels of altruism coupled with patterns in resource utilization (exclusive resources, complementary resources, or essential resources) can influence the evolution of specialism and generalism highlighting the importance of linking evolutionary games to ecological dynamics.

## Mathematical Model and Analysis

### Model outline

#### Dynamics of altruism

We follow Trivers (1985) and define altruism in terms of social behaviors that confers a benefit with a cost. In doing this, we avoid the necessity to model more complex social interactions involving kin interactions and different levels of relatedness. We extend a hawk-dove game (Maynard–Smith 1982) to account for the utilization of resources by two strategies (cooperative [*C*] and selfish [*S*]). This is a useful way to explore evolutionary games that involve strategies that either completely monopolise a resource (selfish individuals—hawks) compared to those that cooperate and share a resource (cooperative individuals—doves). The proportion of altruists (*x*) in a consumer population of size *M* is determined from this evolutionary game and the appropriate cost–benefit (payoff) matrix *V* when two strategies (1 and 2) meet is then defined as (Maynard–Smith 1982):

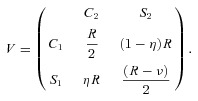
1

Here, *R* is the fixed strategy payoff, η is the probability that selfish individuals monopolize the resource and ν is the cost of squabbling over resource. The change in the proportion of altruists (*x*) through time is given by the following (replicator) equation:

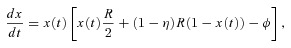
2
where φ is the average population strategy:

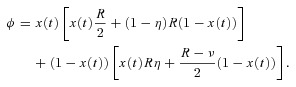
3

#### Ecological dynamics of specialists

The general ecological dynamics for a resource (*N*)—consumer (*M*) interaction describe the population-level consequences when the consumer can feed on a number of different resources. This sort of framework allows the ecological dynamics of specialism to be explore and mathematically is described by:

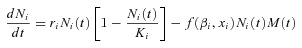
4

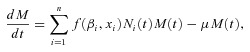
5
where *r*_*i*_ is the growth rate of the *i*th resource, *K*_*i*_ is the carrying capacity of the *i*th resource, µ is the consumer death rate, and *f*(β_*i*_, *x*_*i*_) is the resource utilization function (see below).

#### Linking evolutionary and ecological dynamics

We link the evolutionary and ecological dynamics through the resource utilization function (*f*(β_*i*_, *x*_*i*_)). A consumer’s ability to sequester and utilize a resource is dependent on two processes: the resource uptake process (β_*i*_) and the proportion of consumers that act altruistically (*x*_*i*_) on the *i*th resource. These resource utilization functions can be described in three general ways: concave, linear, or convex:

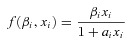
6

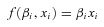
7

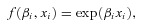
8
where β_*i*_ is the resource consumption rate, *a*_*i*_ is a constant, and 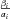 determines the asymptote associated with the concave expression (Equation [6]). These functions have different cost implications (based on the shape of the function) for the effects of altruism when consumers feed on a single versus multiple resources. Furthermore, while the proportion of altruisms determine the resource utilization function, it is important to note that the size of the resource (*N*_*i*_) or consumer (*M*) populations do not feedback directly to affect the proportion of altruists. By making this assumption, we focus on the evolution of specialism and generalism and not on the effects of population size on the evolution of altruism. Below we explore both the biological implications of our chosen resource functions (Equations [6–8]) and the potential for coexistence amongst specialists with these resource utilization strategies.

#### Ecological dynamics of generalists

The evolution of generalism is explored on resources that differ in their impact on consumer populations: resources can be directly substitutable or complementary (essential) (Leon and Tumpson 1975; Tilman 1982). The ecological effects of these different types of resources have been well developed but the consequences of social behaviors on the potential for the evolution of generalist strategies have not previously been considered. The population dynamics of consumers on resources that are entirely substitutable are described by:

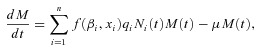
9
where *q*_*i*_ is the preference for the *i*th resource and resources are (linearly) substitutable—that is as preference for one resource goes down the preference for a second resource increase (and both resources have equal effects on population growth).

On complementary (essential) resources (at least), two resources are required for growth. However, while both resources are consumed (hence generalism) only one, the essential limiting resource, determines population growth (Leon and Tumpson 1975). To explore the evolution of generalism on complementary resources, consumption of these resources must differ among strategies. If competition is for the *same* complementary resource then this is primarily evolution of specialism (see above). The dynamics of consumers on complementary resources are determined by:

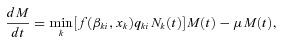
10
where min_*k*_[*f*(β_*ki*_, *x*_*k*_)*q*_*ki*_*N*_*k*_(*t*)] is the minimization function across *i* resources and *q*_*ki*_ is the consumption rate of the *i*th resource by the *k*th strategy.

### Analysis

The analysis of the model proceeds by coupling the evolutionary game (Equation [1]) and the appropriate resource–consumer interaction (Equations [4] and [5] for specialists, Equations [4] and [9] for generalists on substitutable resources, and Equations [4] and [10] for generalists on complementary resources), from which an evolutionary invasion approach can be used to explore both the origins and maintenance of specialism and generalism under different levels of altruism. We derive evolutionary invasion matrices under the different assumptions associated with the resource–consumer dynamics and obtain the invasion criteria under a weak selection limit (see Supporting information). We relax this weak selection assumption and explore the conditions for coexistence of different resource specialist strategies and hence the factors affecting the maintenance of polymorphisms. By evaluating the reciprocal invasion conditions under the different resource responses (Equations [6–8]) where mutations are finite (and sometimes small), we determine when alternative strategies will evolve and replace existing strategies or promote coexistence.

## Results

### Evolution of resource specialization

A strategy is a specialist strategy if it only has access to a single limiting resource. Under linear resource consumption rate (*f*(β_*i*_, *x*) =β_*i*_*x*_*i*_), the population-level growth of a novel rare specialist consumer strategy (*x*_*i*_, *M*_*i*_) defines the success of the strategy (see Supporting information). A novel consumer strategy specializing on a single resource (N*) will invade if its overall net growth rate is greater than zero (see Supporting information) and this occurs if both the following inequalities hold:

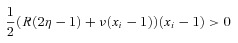
11

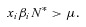
12

The first criterion states that specialization is favored provided the strategy for altruism grows. Within the invading strategy, a proportion of individuals must act altruistically to sequester the necessary level of resource. The second criterion is a standard ecological expectation that requires that specialization is favored if the consumer’s resource consumption rate exceeds the consumer death rate. Both evolutionary and ecological processes have to operate to allow the evolution of resource specialization.

#### Evolution of specialism

**Figure 1:**
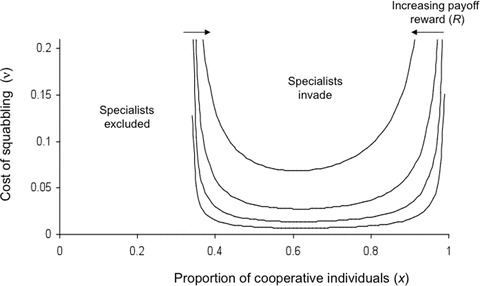
Invasion contours for specialists as a function of costs associated with squabbling (ν) for a resource and the proportion of altruists in a strategy (*x*). Evolution of specialists occurs inside the boundaries. Threshold levels of altruism above which specialization evolves are dependent on increasing levels of payoff reward (*R*).

Evolutionary invasion contours for the evolution of specialists on a single resource as a function of the costs associated with squabbling for the resource (ν) and the proportion of altruistic individuals are shown in Figure 1. These invasion contours show a number of key features associated with this evolution of resource specialization. First, there is a threshold value of altruism below which specialization on a resource cannot occur. This threshold has to be much greater than zero and suggests that specialism cannot occur if selfishness prevails. Second, altruism is expected when (from Equation [1]):

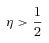
13

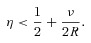
14

In populations comprised principally of selfish individuals, altruism occurs if 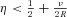 and selfishness is not an evolutionarily stable strategy (ESS). Similarly, altruism is not an ESS as selfish strategies spread when 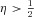. These criteria lead to a bounded region in which altruism is expected to occur—hence, the U-shaped pattern in Figure 1. Finally, increasing the payoff and hence reducing the difference between the costs of squabbling and the cost/benefits associated with a strategy restricts the region of parameter space in which specialization evolves (this is similar to the outcome associated with a hawk-dove game) as 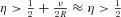.

#### Ecological coexistence of specialists

**Figure 2:**
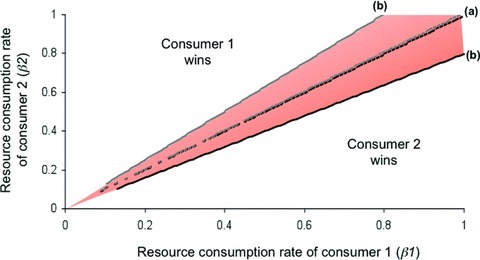
Boundaries of coexistence (in terms of resource consumption rates) for specialists competing for a single resource under (a) linear and (b) concave resource utilization response curves (Equations [6–8]). Particular types of cooperative interactions (concave responses) broaden the regions of coexistence. Increasing levels of resource consumption rates (β) is also more likely to favor the coexistence of specialists. (Shaded area denotes the region of coexistence).

Determining the potential for coexistence of specialists and hence the maintenance of polymorphisms requires evaluating the mutual invasion conditions of phenotypic strategies from rare. These conditions are determined from the strategy invasion matrix and the resulting fitness function (see Supporting information). The effects of the resource utilization (*f*(β_*i*_, *x*_*i*_)) function can affect the patterns of coexistence and evolution (Equations [6–8]). Under the linear resource function (Equation [7]), no coexistence is expected and novel strategies will replace existing strategies depending on the invasion criteria (Equations [11] and [12]). Convex resource utilization (the effects of the proportion of altruists on resource consumption—Equation [8]) introduces additional ecological costs that lead to lower levels of resource consumption (than the linear response) and hence inhibit any potential for coexistence. In contrast, concave functions (Equation [6]) lead to additional benefits of altruism (greater than simple linear resource consumption) and increase the potential for coexistence (Fig. 2).

### Evolution of resource generalists

#### Substitutable resources

**Figure 3:**
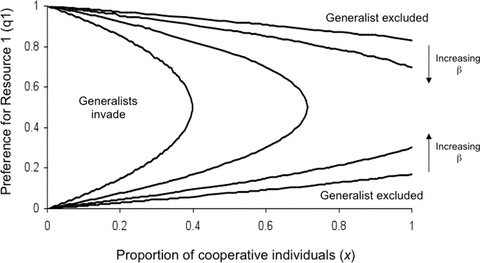
Invasion contours for generalists to evolve on substitutable resources. Generalists are less likely to evolve when preference for a resource is high and the proportion of altruists is also high (which leads to resource specialism). Increasing levels of resource consumption rates (β) are also less likely to favor the evolution of generalism.

Invasion contours (Fig. 3) associated with the fitness condition (see Supporting information) for the evolution of generalists on substitutable resources reveal that there is a threshold level of altruism below which generalism is favored (such that there is no total preference for a single resource). Generalist strategies are expected as altruism becomes rarer. This threshold level declines (altruism is more important in the evolution of specialism) as resource consumption rates increase (β_*i*_). Hence, the region favoring the evolution of generalism is narrowed. More generally, both ecological (preferences for resources, resource consumption rates) and evolutionary (proportion of altruists) factors determine the evolution of generalism under these resource conditions.

#### Complementary resources

**Figure 4:**
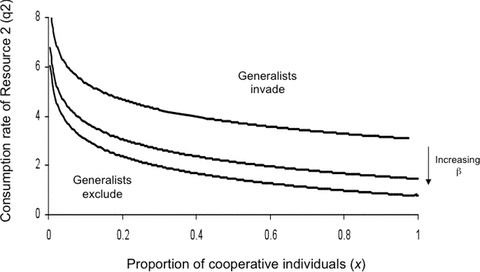
Invasion contours for generalists to evolve when feeding on complementary resources. Generalists feeding on complementary resources are more likely to evolve (invade) as the proportion of cooperative individuals increases. Increasing levels of resource consumption rates (β) are also more likely to promote the evolution of generalists.

Under complementary resources, invasion contours (Fig. 4) associated with the fitness condition (see Supporting information) show that as altruism increases and the level of resource consumption declines the evolution of generalism on complementary resources is more likely. This occurs as each strategy has an essential resource (which is a degree of specialization) that determines population growth and fitness and hence the impact of the social behavior on the evolution of the strategy. Increases in the rate at which the *i*th resource (which is the essential resource) is found (β_*ki*_) increases the evolution of generalism. Finally, there is an indirect (ecological) effect between competitors for complementary resources that drives the patterns affecting the evolution of generalism. While resource consumption of noncomplementary resources does not contribute to population growth of consumer, consumption does affect resource abundance. For instance, the equilibrium abundance of a nonessential resource is (for a two resource system—single consumer system) is:

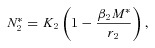
15
where M* is the equilibrium abundance of the consumer:

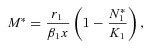
16
which is affected by the density of the complementary resource *N*_1_ (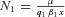). Alterations in the density of complementary resources affect consumers with consequences for noncomplementary resources and hence the evolutionary invasion of novel generalist strategies.

## Discussion

The evolution of social behaviors is often explained by relatedness, the probability of encounter, reputation, and/or local neighbor structure (Haldane 1955; Hamilton 1964a,b; Bourke 2011), where the benefits of the social behavior outweigh the costs of performing it. These sorts of explanations have revolutionized our understanding of the occurrence of altruism and have focused research attention on the exact social and ecological conditions under which these sorts of behaviors might be expected to evolve (e.g., Dugatkin 1997). While it is appreciated that interactions among conspecific individuals affect the resource acquisition, the potential to affect resource–consumer interactions and the evolution of resource specialism remains less clearly understood. Here, we have shown how altruism can affect the evolution of resource use. Using an evolutionary dynamic approach, we have elucidated the conditions under which we might expect resource generalism and specialism to evolve. In particular, we show that a threshold condition exists below which resource specialism is not expected to evolve and that this threshold is highly dependent on the payoffs received for adopting the strategy (*R* in Equation [1]). Similarly, threshold conditions on the level of cooperation determine when generalism is expected: if resource preferences are weak and consumption rates are low, generalists are expected to evolve.

Ecological dynamics are affected by the social behavior. The evolution of generalism in the presence of complementary resources leads to an indirect ecological interaction. Positive changes in the density of complementary resources affect consumers with negative consequences for noncomplementary resources (Equations [15] and [16]) and vice versa. The availability of the essential limiting resource and the prevalence of cooperation will have consequences for the evolution of novel generalist strategies. This sort of indirect interaction is an apparent competitive effect, where a consumer accesses multiple shared resources (Holt 1977; Bonsall and Hassell 1997; Chaneton and Bonsall 2000) and the outcomes are influenced not only by the resource–consumer interaction but also by the level of altruism. The consequences of these sorts of feedbacks between ecological and social interactions warrant further attention.

Furthermore, patterns of coexistence are also likely to be influenced by the degree of altruism. We explored this using different resource utilization functions (Equations [6–8]). The linear relationship describing the response between resource utilization and proportion of altruists within a population can be thought of as a beneficial social behavioral strategy (sensu Trivers 1985). The alternative functional responses such as interactions, described by the concave function, lead to a greater proportion of a resource being accessed for a given level of altruism. The evolution of specialism might be more likely when these sorts of social behaviors operate. In contrast, competitive interactions diminish the returns from altruistic interactions, limiting the potential for coexistence, and promoting the evolution of generalism. Competition can limit social interactions (Platt and Bever 2009) but this crucially depends on population structure. Population-level effects where the density dependence operates locally (Levene 1953; Dempster 1955; Karlin and Campbell 1981) and resources are subdivided in a fine-grain environment (Levins 1962, 1968) are more likely to lead to strong competitive interactions restricting coexistence and favoring the evolution of generalists. However, this will be influenced by the timing of density dependence with respect to other life-history effects (such as dispersal or heterospecific competition). Local density-dependent processes will reduce individual fitness in larger groups as *per capita* changes in population size will be larger. If dispersal happens before density dependence then the effects on fitness may be reduced and this will affect the evolution of specialism by affecting the proportion of altruists in a population.

Resource partitioning is well established as a mechanism by which multiple consumers can coexist (Miller 1967). The availability of exclusive resource rights diminishes the degree of interspecific competition and can favor coexistence. This effect has been widely demonstrated in a diverse range of taxa under different ecological scenarios (e.g., Dykhuizen and Davies 1980; Hassell and May 1986; Gherardi 1990; Bonsall et al. 2004a; Dammhahn and Kappeler 2008; Hunt and Bonsall 2009). Competitive exclusion is also influenced by the ecological dynamics of the resource–consumer interaction (Armstrong and McGehee 1980). Temporal fluctuations in resources affect the (nonlinear) competitive coefficients and promote coexistence as the average resource abundance patterns are significantly different from the equilibrial resource level (Armstrong and McGehee 1980). Underpinning this are alterations in the number of limiting processes affecting patterns of coexistence (Levin 1970; May 1973). At a broad mechanistic level, altruistic interactions are likely to extend the factors limiting populations and promote coexistence (Fig. 4) but understanding more fully how resource competition and social behavior drive resource cycles by influencing ecological limitation and regulation necessitates a closer interplay between evolutionary game theory, ecological dynamics, and experiments.

Variability in resource productivity will affect the degree of competition and hence social interactions both of which are clearly dependent on the spatial scale of interactions (e.g., Nowak and May 1992; Kelly 1994). Arguably, social interactions are less likely when the scale of altruism equals or exceeds the scale at which group-size regulation occurs (Taylor 1992). However, the implications of these findings for the evolution of resource specialism or generalism are not obviously clear as this will depend on how habitats are utilized. More importantly, any environmental heterogeneity that affects the predictability of resources will have consequences for the population structures, local competition and cooperation and, hence the evolution of specialism. If environments are homogeneous and resources are predictable, it is anticipated that this will promote a high level of cooperation leading to resource specialization. Conversely, when environments are heterogeneous and resources are more unpredictable, generalism will be expected. To some degree, these predictions are borne out by empirical examples. Mixed resource utilization by microorganisms is known to be dependent upon their concentration (Harder and Dijkhuizen 1982). When substrates are present in high concentrations that do not limit growth then the resource that ensures highest growth is preferentially utilized: specialism is favored. When mixed resources are available that are growth limiting, simultaneous use of multiple sources (generalism) is the expected response (Harder and Dijkhuizen 1982). As far as we know as of yet, there remain no empirical demonstrations of the role of altruistic behaviors driving resource specialism and generalism but given this extensive information on mixed substrate utilization it is clear that there are some extremely amenable empirical systems for testing this hypothesis.

As noted, coexistence of multiple specialist strategies requires life-history trade-offs. The evolutionary mechanisms for this diversification and the maintenance of diversity across a resource require disruptive selection to maintain limiting similarity (Bonsall et al. 2004b). These processes of diversification (through adaptive radiations; the emergence of ecological diversity from a single lineage) provide evidence for the evolution of resource specialism. For instance, the radiation of Darwin’s Galapagos finches is the archetypal adaptive radiation: the origin of new species of finch could only persist if sufficiently distinct and adapted to different parts of the niche hypervolume, restricting their ecology, and leading to specialization (Lack 1947; Grant 1986). More recently, broader phylogenetic analysis (Schluter 2000) confirms this sort of pattern of increasing specialization but also highlights that it might not necessarily be universal. A key driver in these adaptive radiations and driving resource specialization is life-history trade-offs (Futuyma and Moreno 1988). Recent work on adaptive radiations and resource specialization has grown rapidly (Schluter 2000) particularly in microbial systems (Kassen and Rainey 2004; MacLean 2005). Evolutionary trade-offs have been implicated in patterns of resource utilization (Pfeiffer et al. 2001; Kreft and Bonhoeffer 2005; MacLean and Gudelj 2006; Gudelj et al. 2007) and social behaviors are thought to affect certain trade-offs such as the link between the rate and yield of adenosine triphosphate (ATP) production. Pfeiffer et al. (2001) argue that high ATP production rate but low yield may be selectively advantageous on shared resources but social interactions in spatially structured environments can modulate this effect to favor low rate high yield strategies. MacLean and Gudelj (2006) provide empirical support for these theoretical predictions but the scale of resource heterogeneity (in particular in structured environments) may mask phenotypic trade-offs such as the link between growth and competitive ability (Velicer and Lenski 1999). The genetic basis for the evolution of specialization through trade-offs requires antagonistic pleiotropy (Elena and Lenski 2003; Dykhuizen and Dean 2004). As noted, this might be manifest as a direct trade-off where performance on one resource is negatively correlated with performance on a second resource. Alternatively, pleiotropic effects might arise through greater fitness increases on one resource compared to that on a second resource. Further mechanisms that might lead to the evolution of specialization include genetic drift, evolutionary cataclysms, or the accumulation of mutations (of different size effects) (Bidder 1930; Elena and Lenski 2003; Dykhuizen and Dean 2004). While these mechanisms do not preclude selection for altruists, understanding the hierarchy of mechanisms that facilitate the evolution of specialism and generalism is amenable to further theoretical and empirical analysis.

In summary, using an evolutionary dynamic approach (linking evolutionary games to nonlinear ecological dynamics), we have elucidated the conditions under which we might expect resource generalism and specialism to evolve and be maintained when altruism operates. Separating out the origins from the maintenance of traits is a critical dichotomy in evolution that is too often conflated. Our approach based on a natural selection framework of the dynamics of a cost–benefit interaction amongst altruists is an appropriate way to develop predictions on the outcome of resource competition and specialization. Alternative approaches (often under limited scenarios of weak selection and additive cost–benefit effects) may be appropriate for assessing the maintenance of social behavioral traits but none of these can be adequately extended for exploring the ecological dynamical implications of trait evolution. These caveats notwithstanding we have shown that threshold conditions in the levels of altruisms coupled with resource–consumer interactions can be important drivers in the evolution of specialism and generalism.
