# Supplementary material for: Altruism and the evolution of resource generalism and specialism
Source: Ecol Evol. 2012 Mar;2(3):515–24. doi: 10.1002/ece3.206 (PMC3399141; doi:10.1002/ece3.206)
Supplement: Supplementary file 1 [file ece30002-0515-SD1.pdf]

Supporting Information - Evolutionary Analysis

An evolutionary invasion approach is used to explore the consequences of cooperation on the evolution of resource utilisation. This approach is similar to adaptive dynamics (Metz et al. 1992; Geritz et al. 1998; Waxman and Gavrilets 2005) in which ecological and evolutionary timescales are distinct and mutants are rare as they invade a resident population. In contrast to the adaptive dynamic approach, this invasion approach considers evolutionary conditions when mutations are finite and only sometimes small (Bonsall and Mangel 2009). Our analysis begins by defining an ecological model and thus creating the background in which the evolution of can resource utilisation occur. We establish using the evolutionary game and the ecological dynamics (eqn 1-8). Next an appropriate measure of fitness is defined. When strategies are rare and ecological processes such as density dependence operate, this measure of fitness is the per capita population-level growth rate (e.g.,$\frac{1}{N}\frac{dN}{dt}$). Finally, changes in fitness with respect to specific life-history traits allow both the origin and maintenance of resource utilisation strategies to be explored.

A Evolution of Resource Specialization

The dynamics of the resource utilisation are dependent on both the proportion of cooperators in a population given by the replicator equation (equation 2) and the dynamics of the ecological interaction between specialist consumers and a resource (described by equations 4-5). Under linear resource consumption rate ($f(\beta_i, x) = \beta_i x_i$) (equation 7), the population-level growth (fitness) of a novel rare consumer strategy (x_i, M_i) can be found linearizing the equations (eqn

2, 4-5) around the equilibirum resource density (N^*), finding the partial derivatives for the novel invading strategy and then taking the determinant of the resulting Jacobian matrix (Pielou 1977):

$$\mathbf{A} = \begin{pmatrix} \lambda - x_i\beta_i N^* + \mu & -\beta_i N^* M_i \\ 0 & \lambda - \frac{1}{2}\left(R(2\eta - 1)(2x_i - 1) + \nu(1 - 4x_i + 3x_i^2)\right) \end{pmatrix} \quad \text{(A.1)}$$

where λ is the measure of fitness and N^* is the equilibrium abundance of the resource in the absence of the novel consumer (M_i) strategy. The entries in this matrix are the partial derivatives associated with the way in which the novel consumer strategy utilises the resource and the dynamics of the cooperators within this novel strategy. The fitness measure (λ) is obtained by solving the characteristic equation of $\mathbf{A}$ which is given by:

$$\lambda^2 - tr(\mathbf{A})\lambda + det(\mathbf{A}) \quad \text{(A.2)}$$

where in a simple two-by-two matrix $\mathbf{A} = \begin{pmatrix} a & b \\ c & d \end{pmatrix}$, the $tr(\mathbf{A}) = a + d$ and $det(\mathbf{A}) = ad - cb$. If the difference between mutant and resident strategy is small such that selection is weak then λ is sufficient small and terms involving λ^2 are negligible so fitness is then:

$$\lambda \approx \frac{det(\mathbf{A}}{tr(\mathbf{A})} \quad \text{(A.3)}$$

$$\lambda \approx -\frac{\left(R(2\eta - 1)(2x_i - 1) + \nu(1 - 4x_i + 3x_i^2)\right)(x_i\beta_i N^* - \mu)}{R(2\eta - 1)(2x_i - 1) + \nu(1 - 4x_i + 3x_i^2) + 2(x_i\beta_i N^* - \mu)}. \quad \text{(A.4)}$$

Contours of fitness associated with resource specialization based on equation (A.4) are illustrated in Figure 1.

B Evolution of Resource Generalism

The evolution of resource generalism proceeds in a similar way by defining both the ecological dynamics and the measure of fitness and then evaluating the changes in fitness.

a) Substitutable Resources

On substitutable resources, the dynamics of the consumers are determined by equation (9) where a preference for particular resources leads to linear increases or decreases in resource utilisation. Resources are then entirely substitutable. As before the dynamics of cooperation is determined by the replicator equation (eqn 2). Analysis proceeds by modifying the appropriate terms in the matrix (equation A.1). In particular, (when there are two substitutable resources) the partial derivatives associated with the ecological dynamics are now:

$$\mathbf{A} = \begin{pmatrix} \lambda - \frac{x_i \beta_1 N_1^*}{q_1} - \frac{x_i \beta_2 N_2^*}{q_2} + \mu & -M\left[\frac{\beta_1 N_1^*}{q_1} + \frac{\beta_2 N_2^*}{q_2})\right] \\ 0 & \lambda - \frac{1}{2}\left(R(2\eta - 1)(2x_i - 1) + \nu(1 - 4x_i + 3x_i^2)\right) \end{pmatrix} \tag{B.1}$$

where q_j is the preference for the jth resource. Again taking the determinant of this matrix (B.1) (under linear consumption - equation 7) provides a measure of the fitness of the generalist strategy on substitutable resource. Applying the weak selection limit yields:

$$\lambda \approx \frac{\left(R(2\eta - 1)(2x_i - 1) + \nu(1 - 4x_i + 3x_i^2)\right)\left(x_i(q_2\beta_{1i}N_1^* + q_1\beta_{2i}N_2^*) - \mu q_1 q_2\right)}{2\mu q_1 q_2 - 2N_1^* q_2 x_i \beta_{1i} - q_1\left[q_2\left(R(2\eta - 1)(2x_i - 1) + \nu(1 - 4x_i + 3x_i^2)\right) + 2N_2^* x_i \beta_{2i}\right]}. \tag{B.2}$$

Figure 3 illustrates the fitness contours associated with the evolution of resource utilization on substitutable resources.

b) Complementary Resources

On complementary (essential) resources the consumer dynamics are specified by equation (10). Modifying the terms in the invasion matrix (equation A.1) leads to the following set of partial derivatives:

$$\mathbf{A} = \begin{pmatrix} \lambda - \frac{x_i\beta_2 N_2^*}{q_2} + \mu & -M\frac{\beta_2 N_2^*}{q_2} \\ 0 & \lambda - \frac{1}{2}\left(R(2\eta-1)(2x_i-1) + \nu(1-4x_i+3x_i^2)\right) \end{pmatrix}. \tag{B.3}$$

Solving the characteristic equation from the determinant of equation B.3 under the weak selection limit gives the fitness of a generalist strategy on two complementary resources. This yields:

$$\lambda \approx -\frac{\left(R(2\eta-1)(2x_i-1) + \nu(1-4x_i+3x_i^2)\right)(x_i\beta_{2i}N_2^*) - \mu q_2)}{q2\left[R(2\eta-1)(2x_i-1) + \nu(1-4x_i+3x_i^2)\right] - 2(x_i\beta_{2i}N_2^* + \mu q_2)}. \tag{B.4}$$

Figure 4 illustates the fitness contours associated with this expression.

References

Bonsall, M.B. and M. Mangel. 2009. Density dependence, lifespan and the evolutionary dynamics of longevity. Theor. Pop. Biol. 75: 46-55.

Geritz, S.A.H., E. Kisdi, G. Meszena and J.A.J. Metz. 1988. Evolutionary singular strategies and the adaptive growth and branching of the evolutionary tree. Evol. Ecol. 12: 35-57.

Metz, J.A.J., R.M. Nisbet and S.A.H. Geritz. 1992. How should we define fitness for general ecological scenarios. Trends Ecol. Evol. 7: 198-202.

Pielou, E.C. 1977. Mathematical Ecology. John Wiley & Sons, New York.

Waxman, D. & S. Gavrilets. 2005. 20 questions on adaptive dynamics. J. Evol. Biol. 18: 1139-1154.
